# Supplementary figures and images for: TET1 may contribute to hypoxia-induced epithelial to mesenchymal transition of endometrial epithelial cells in endometriosis
Source: PeerJ. 2020 Sep 15;8:e9950. doi: 10.7717/peerj.9950 (PMC7500323; doi:10.7717/peerj.9950)

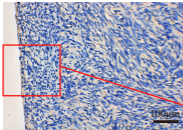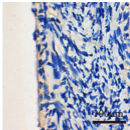

Supplement: Figure S2 [file peerj-08-9950-s002.pdf]
